# Proximal renal tubular acidosis mediated by mutations in NBCe1-A: unraveling the transporter's structure-functional properties

**DOI:** 10.3389/fphys.2013.00350

**Published:** 2013-12-19

**Authors:** Ira Kurtz, Quansheng Zhu

**Affiliations:** ^1^Division of Nephrology, David Geffen School of Medicine, UCLALos Angeles, CA, USA; ^2^Brain Research Institute, UCLALos Angeles, CA, USA

**Keywords:** NBCe1, bicarbonate, carbonate, transport, proximal tubule, kidney, proximal renal tubular acidosis

## Abstract

NBCe1 belongs to the SLC4 family of base transporting membrane proteins that plays a significant role in renal, extrarenal, and systemic acid-base homeostasis. Recent progress has been made in characterizing the structure-function properties of NBCe1 (encoded by the *SLC4A4* gene), and those factors that regulate its function. In the kidney, the NBCe1-A variant that is expressed on the basolateral membrane of proximal tubule is the key transporter responsible for overall transepithelial bicarbonate absorption in this nephron segment. NBCe1 mutations impair transepithelial bicarbonate absorption causing the syndrome of proximal renal tubular acidosis (pRTA). Studies of naturally occurring NBCe1 mutant proteins in heterologous expression systems have been very helpful in elucidation the structure-functional properties of the transporter. NBCe1 mutations are now known to cause pRTA by various mechanisms including the alteration of the transporter function (substrate ion interaction, electrogenicity), abnormal processing to the plasma membrane, and a perturbation in its structural properties. The elucidation of how NBCe1 mutations cause pRTA in addition to the recent studies which have provided further insight into the topology of the transporter have played an important role in uncovering its critically important structural-function properties.

## Introduction

Renal tubular acidosis (RTA) can be divided clinically into proximal RTA (pRTA) caused by defective proximal tubule bicarbonate absorption (Haque et al., 2012), and distal RTA (dRTA) resulting from impaired collecting duct net acid excretion (Battle and Haque, 2012). This review highlights the structure-function abnormalities in NBCe1-A caused by mutations in the transporter that result in autosomal recessive pRTA.

**Figure 1:**
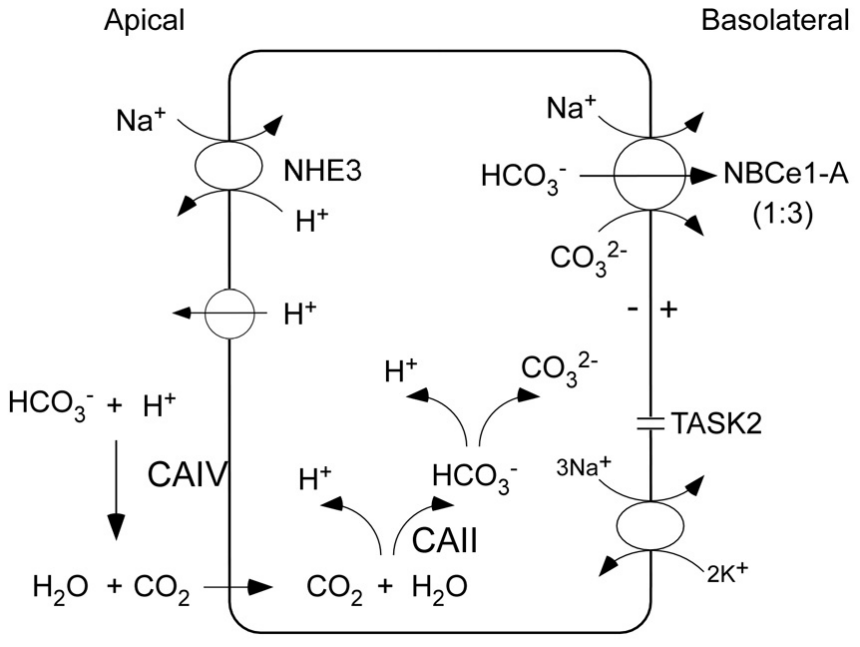
**Coupled apical and basolateral H^+^/base transport in proximal tubule cells mediates trancellular transport of bicarbonate**. The charge transport stoichiometry of NBCe1-A in the human proximal tubule is unknown and is depicted as 1:3. Luminal bicarbonate is initially protonated via NHE3 (and to a lesser extent an apical V-ATPase) and is converted to CO_2_ via a dehydration reaction that is accelerated by GPI anchored CAIV. Dissolved CO_2_ permeates the apical membrane passively through the lipid bilayer (or possibly through AQP1) down its concentration gradient into the cell. In the cytoplasm, the reverse hydration reaction catalyzed by cytoplasmic CAII converts CO_2_ to HCO^−^_3_ which is in equilibrium with CO^2−^_3_. The basolateral Na^+^-K^+^-ATPase coupled with TASK2 K^+^ channels generate the basolateral membrane potential, creating an electrical driving force for NBCe1-A mediated electrogenic Na^+^-base efflux.

Transepithelial bicarbonate absorption in the proximal tubule is an indirect two-step process driven by the coupled transport of the apical Na^+^/H^+^ exchanger NHE3 (and a quantitatively less important apical H^+^-ATPase), in parallel with basolateral electrogenic Na^+^-base transport mediated by NBCe1-A (Figure 1; Boron, 2006; Hamm et al., 2013). According to current concepts, luminal bicarbonate is initially protonated via NHE3 and is ultimately via a dehydration reaction (accelerated by GPI anchored CAIV) converted to CO_2_ which is transferred across the apical membrane down its concentration gradient into the cytoplasm. In the cytoplasm, the reverse hydration of CO_2_ to HCO^−^_3_ and CO^2−^_3_ is catalyzed by cytoplasmic CAII. The basolateral membrane potential generated by the basolateral Na^+^-K^+^-ATPase and TASK2 K^+^ channels (Warth et al., 2004), drive electrogenic Na^+^-base transport via NBCe1-A. Interestingly, mutations in NHE3 have not been documented clinically; mutant CAIV causes retinitis pigmentosa (RP17) without pRTA having been reported in patients (Rebello et al., 2004) nor in mice with targeted CAIV disruption (Shah et al., 2005); and in patients with CAII mutations (discussed in greater detail below) causing a mixed pRTA and dRTA, the pRTA component can be mild suggesting that compensatory processes may be involved (Sly et al., 1985). Of the known proteins that play an important role in transepithelial bicarbonate absorption, only mutations in NBCe1-A that is the focus of this review have been documented to a cause a clinically important defect in proximal tubule transepithelial bicarbonate absorption.

NBCe1 is expressed in various extrarenal organs and accordingly patients with autosomal recessive pRTA due to NBCe1 mutations can be diagnosed clinically (without the need for genetic testing) because of the specific systemic phenotypic abnormalities that include, growth and mental retardation, glaucoma, cataracts, corneal opacities (band keratopathy), basal ganglia calcification, elevated serum lipase and amylase, and enamel defects (Tables 1 and 2; Igarashi et al., 1999; Kurtz, 2013). The loss of NBCe1 in mice results in an even more severe phenotype that includes colonic abnormalities, volume deletion, and decreased survival (Gawenis et al., 2007; Lacruz et al., 2010). Whether heterozygous family members also have subtle defects in proximal tubule bicarbonate transport and/or mild extrarenal (ocular, brain, growth, and enzymatic abnormalities) has not been determined. Interestingly, migraine headaches have been reported in patients with the R510H, L522P, and R881C missense mutations, 2311 delA, and a homozygous C-terminal 65 base-pair deletion (Suzuki et al., 2010). Headaches are hypothesized to be due to abnormal NMDA-mediated neuronal hyperactivity due to ER retained misfolded NBCe1-B in brain astrocytes. Headaches have also been reported in heterozygous family members of a patient with a 65 base-pair C-terminal deletion and the L522P mutation thought to be due to hetero-oligomer formation resulting in ER retention of wild-type transporters (Suzuki et al., 2010; Yamazaki et al., 2013). The possibility that single nucleotide polymorphisms (SNPs) alter the function of NBCe1-A in otherwise normal individuals was examined by Yamazaki et al. who studied the function of 4 SNPs in E122G, S356Y, K558R, and N640I, and reported that the base transport function of K558R was decreased 41-47% (Yamazaki et al., 2011).

**Table 1:** **NBCe1-A pRTA causing mutations**.

**Mutation^a^**	**Classification**	**Location**	**Effect of mutation^b^**
Q29X	Nonsense	N-terminal region	NBCe1-A protein truncation
R298S	Missense	N-terminal region	-mistargeting: apical/basolateral membranes
			-abnormal interaction of the N- terminal region with the cytoplasmic region
S427L	Missense	TM1	-mistargeting: predominant apical membrane
			-abnormal helix packing
			-decreased G_HCO3_
			-impaired I_HCO3_ reversal at –Vm
T485S	Missense	TM3	-altered ion interaction
			-electroneutral transport
G486R	Missense	TM3	Altered ion interaction
R510H	Missense	TM4	Intracellular retention (ER)
W516X	Nonsense	TM4	Truncation of all NBCe1 variants
L522P	Missense	TM4	Intracellular retention (ER)
2311 delA	Frameshift	IL4	Truncation of all NBCe1 variants
A799V	Missense	TM10	-intracellular retention
			-decreased G_HCO3_
			-bicarbonate-independent G_cation_
R881C	Missense	TM12	Intracellular retention (ER)
65 bp-del	Frameshift	C-terminal tail	Intracellular retention (ER)

a *The mutations are numbered according to the NBCe1-A amino acid sequence*.

b *There are no immunocytochemistry studies of mutant NBCe1-A transporters in proximal tubule cells from kidney biopsies in patients with pRTA; biopsies from extrarenal tissues delineating the expression pattern of other mutant NBCe1 transporters is also lacking*.

*G_HCO3_: bicarbonate conductance; G_cation_: cation conductance; I_HCO3_: bicarbonate-dependent current; - Vm: negative plasma membrane voltages*.

*Q29X (Igarashi et al., 2001; Azimov et al., 2008); R298S (Igarashi et al., 1999; Horita et al., 2005; Li et al., 2005 (NBCe1-B-R342S); Chang et al., 2008; Suzuki et al., 2008, 2010; Zhu et al., 2010b); S427L (Dinour et al., 2004; Li et al., 2005; Zhu et al., 2009, 2010b, 2013a); T485S (Horita et al., 2005; Suzuki et al., 2008, 2010; Zhu et al., 2010b, 2013b); G486R (Suzuki et al., 2008, 2010; Zhu et al., 2010b, 2013b); R510H (Igarashi et al., 1999; Horita et al., 2005; Li et al., 2005; Suzuki et al., 2010; Zhu et al., 2010b; W516X Lo et al., 2011; L522P: Demirci et al., 2006; Suzuki et al., 2008, 2010; Zhu et al., 2010b; Yamazaki et al., 2013); 2311 delA (Inatomi et al., 2004); A799V Deda et al., 2001; Horita et al., 2005; Suzuki et al., 2010; Zhu et al., 2010b; Parker et al., 2012; R881C (Horita et al., 2005; Toye et al., 2006; Suzuki et al., 2010; Zhu et al., 2010b); 65 bp-del (Suzuki et al., 2010; Yamada et al., 2011)*.

**Table 2:** **Hereditary^a^ proximal renal tubular acidosis**.

**Gene**	**Protein**	**Inheritance**	**Renal phenotype**	**Extra-renal phenotype**
SLC4A4	NBCe1	Autosomal recessive	pRTA, hypokalemia	Growth defect^b^,^c^; decreased IQ; intracerebral calcification; band keratopathy; glaucoma; cataracts; elevated serum lipase and amylase and enamel defects
CA2	CAII	Autosomal recessive	pRTA^d^, dRTA, hypokalemia	Growth defect; intracerebral calcification; osteopetrosis involving skull, axial skeleton, and long bones with widening of metaphyses
Unknown gene(s)^e^	Unknown	Autosomal dominant	pRTA	Growth defect; colomboma; sub-aortic stenosis; decreased radial bone density; thinner iliac cortices

a *Genetic diseases causing pRTA in the context of other proximal tubule transport defects are shown in Table 3*.

b *Migraine headaches have been reported in patients with the R510H, L522P, and R881C missense mutations, 2311 delA, and a homozygous C-terminal 65 bp-del. Headaches have also been reported in heterozygotes with 65 bp-del and the L522P mutations attributed to a dominant-negative effect*.

c *To what extent the extrarenal phenotype is consequence of systemic acidemia vs. abnormal tissue specific NBCe1 transport is not well understood*.

d *pRTA due to CAII deficiency can be less severe than in patients with NBCe1 mutations and in patients taking CA inhibitors, likely due to compensatory processes in the proximal tubule. In addition, unlike pRTA due to NBCe1 mutations, distal RTA (dRTA) is also present*.

e *Mutations were not found in CAII, CAIV, CAXIV, NBCe1, NHE3, NHE8, NHERF1, and NHERF2, and PAT1(CFEX) (Katzir et al., 2008)*.

**Table 3:** **Genetic causes of pRTA with additional proximal tubule transport abnormalities**.

**Gene**	**Inheritance**	**Protein/function**	**Disease**
CLCN5^a^	X-linked	2Cl^−^/H^+^ exchanger	Dent's disease 1
OCRL1	X-linked	PIP2 5-phosphatase	Dent's disease 2
ATP7B	Autosomal recessive	Cu^++^ transporting ATPase beta peptide	Wilson's disease
GALT	Autosomal recessive	Galactose-1-phosphate uridylyltransferase	Galactosemia
ALDOB	Autosomal recessive	Aldolase B	Hereditary fructose intolerance
FAH	Autosomal recessive	Fumarylacetoacetase	Tyrosinemia type I
CTNS	Autosomal recessive	Cystinosin	Cystinosis
OCRL1	X-linked	PIP2 5-phosphatase	Lowe's syndrome
SLC2A2	Autosomal recessive	GLUT2	Fanconi-Bickel syndrome
MMAB	Autosomal recessive	Methylmalonyl CoA mutase	Methylmalonic acidemia
PC	Autosomal recessive	pyruvate carboxylase	Pyruvate carboxylase deficiency
ARSA	Autosomal recessive	Arylsulfatase A	Metachromatic leukodystrophy
Complex IV^b^	N/A	Cytochrome C oxidase	Cytochrome C oxidase deficiency

a *Approximately 60% of patients have mutations in the CLCN5 and 15% of patients have mutations in the OCRL1 gene. Mutations in OCRL1 also cause Lowe's syndrome*.

b *Complex IV or Cytochrome C oxidase is the terminal enzyme in the respiratory chain and potentially involves mutations in several nuclear- and mitochondrial-encoded genes*.

**Figure 2:**
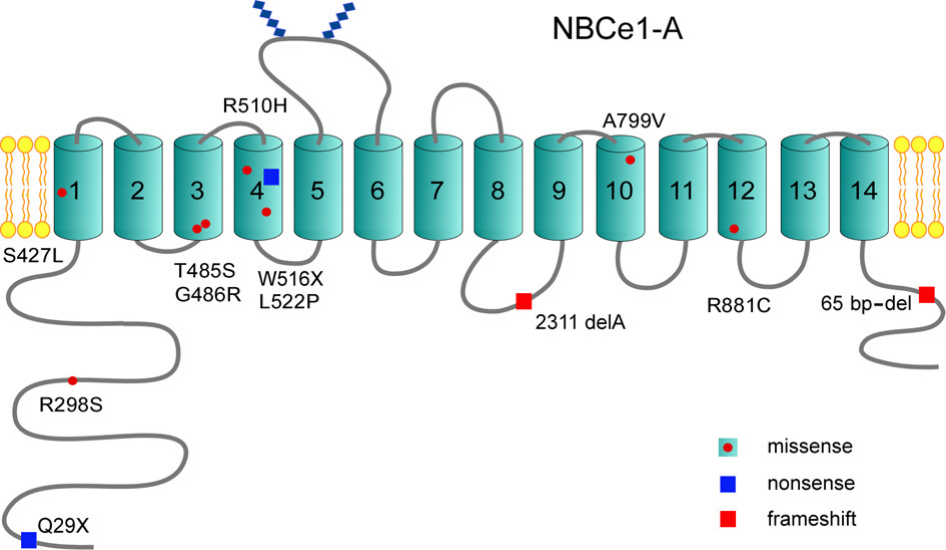
**Structural topology of NBCe1-A: The NBCe1-A variant has been the most thoroughly studied and is depicted**. Thus far, 12 NBCe1 mutations have been reported that include 8 missense mutations, 2 nonsense mutations, and 2 frameshift mutations. Of the reported missense mutations, the majority are localized to the transmembrane region. All NBCe1 variants share the identical transmembrane region where ion transport is mediated, and have a large cytoplasmic N-terminal region with a shorter cytoplasmic C-terminal tail.

Mutations throughout the transporter including 2 nonsense mutations (Q29X, W516X), a frameshift deletion at nucleotide 2311 (2311 delA), a C-terminal 65 base-pair deletion from exon 23 to intron 23 (predicted to truncate the C-terminus), and 8 missense mutations are reported (Table 1; Figure 2; Zhu et al., 2010b). Based on the most recent topology of NBCe1 (Zhu et al., 2010b) other than the cytosolic Q29X, R298S, and C-terminal 65 base-pair deletion mutations, and the 2311 delA frameshift stop codon mutation in intracellular loop 4 (IL4), the remaining pRTA mutations reside in the transmembrane region. This finding is clinically relevant since the human *SLC4A4* gene (Abuladze et al., 2000) encodes 3 variants (-A, -B, and -C) with additional variants (-D and -E) having been reported in mouse (Liu et al., 2011). All 5 mammalian NBCe1 variants have the identical transmembrane region differing in their N- and C-terminal sequences. NBCe1-A/-B/-C have been shown to mediate electrogenic Na^+^-base transport (McAlear et al., 2006) but differ in their tissue expression, intrinsic activity, and regulation.

NBCe1-A is predominantly expressed in S1 and S2 proximal tubules where it mediates basolateral Na^+^-base efflux contributing to the reabsorption of ~80% of the filtered bicarbonate (Skelton et al., 2010). NBCe1-B was originally cloned from pancreas and unlike NBCe1-A and NBCe1-C is widely expressed in multiple organs with a unique N-terminus wherein 85 aa replaces the 41 aa in NBCe1-A) (Abuladze et al., 1998). NBCe1-C originally cloned from rat brain has a unique C terminus (61 aa replaces 46 aa in NBCe1-A or B) ending in a type I PDZ-binding motif (McAlear et al., 2006). NBCe1-D and NBCe1-E transcripts more recently identified in mouse reproductive tissues are otherwise identical to NBCe1-A and NBCe1-B respectively, and within the cytosolic N-terminus lack a predicted nine amino-acid sequence (Liu et al., 2011). A long-standing unanswered question is whether the extrarenal symptoms in patients with pRTA result from defective locally expressed NBCe1 protein variants, or as a consequence of systemic acidemia due to pRTA *per se*.

## Overall NBCe1 topology

NBCe1-A exists as a homodimer in the plasma membrane, of which each monomer functions independently (Kao et al., 2008; Sergeev et al., 2012). Extensive topological analysis has shown that each NBCe1-A monomer contains a large N-terminal cytoplasmic region, a plasma membrane embedded transmembrane region, and a short C-terminal cytoplasmic tail (Figure 2) (Zhu et al., 2010a,b, 2013a). Unlike the highly aqueous exposed C-terminal cytoplasmic tail, the N-terminal cytoplasmic region is modeled to form a domain structure and is tightly folded (Zhu et al., 2013a). The oligomerization of the N-terminal region appears to be pH and/or bicarbonate dependent (Gill, 2012). The transmembrane region has been determined to contain 14 lipid embedded transmembrane helices (Zhu et al., 2010a,b). TM5 and 6 bracket a large extracellular loop 3 that contains two glycosylated sites (Choi et al., 2003). Although NBCe1 variants differ in the sequence of their N-terminal regions and/or C-terminal tails, they all are predicted to share the same topographic structure as NBCe1-A.

## The N-terminal cytoplasmic region (AID, ASD; regulation by IRBIT, PIP_2_, Mg^2+^; pRTA Q29X and R298S mutations)

The unique N-terminus of NBCe1-A functions as an autostimulatory domain (ASD) because of its ability to stimulate transporter function through an unknown mechanism (McAlear et al., 2006). The autostimulation has been hypothesized to be mediated by specific residues in the N-terminus of NBCe1-A which can potentially interact with a region(s) in the ion permeation pathway. Recent evidence suggests that that the N-terminal cytoplasmic region of NBCe1-A can interact with the transmembrane region (Zhu et al., 2013a). Studies of the cytoplasmic N-terminally localized R298S pRTA mutation indicate that it resides in an aqueous inaccessible tightly folded region that forms a “HCO^−^_3_ tunnel” whose structure is disrupted in the mutant transporter (Igarashi et al., 1999; Horita et al., 2005; Chang et al., 2008; Suzuki et al., 2010; Zhu et al., 2010b). By preventing the putative interaction of the N-terminus with the transmembrane region, the efficient delivery of base to the ion permeation pathway in the transmembrane region may be perturbed. An additional mechanism is suggested from studies of the NBCe1-A-R298S and NBCe1-B-R342S mutants expressed in MDCK cells. Both mutants were mistargeted to both the apical and basolateral membranes (Li et al., 2005; Suzuki et al., 2008) suggesting that targeting signal(s) may also be affected. The nonsense Q29X mutation in NBCe1-A is unique in that it affects the expression of the NBCe1-A variant specifically (Igarashi et al., 2001; Azimov et al., 2008). The mouse NBCe1-D variant which shares the same N-terminus if expressed in humans would be predicted to also be involved (Liu et al., 2011). There are currently no reported NBCe1 mutations that are specific for other NBCe1 variants. Previous studies in HEK 293-H cells expressing the NBCe1-A-Q29X mutant have shown that in the presence of the aminoglycoside G418 which induces ribosomal read-though, full-length functional NBCe1-A protein can be produced (Azimov et al., 2008).

Unlike NBCe1-A, the unique N- terminus of the -B and -C variants (and possible the -E variant) have an autoinhibitory domain (AID) that inhibits NBCe1 transport (McAlear et al., 2006; Lee et al., 2012). Studies of heterologously expressed NBCe1 variants in cultured cell and *Xenopus* oocyte expression systems have revealed the regulatory mechanisms involved. Amino acids 37–65 (a positively charged motif) in the N-terminus of NBCe1-B (absent in NBCe1-A and NBCe1-D) mediate the interaction of NBCe1-B with IRBIT and PIP_2_ (Hong et al., 2013). IRBIT activates NBCe1-B by preventing the inhibition by AID through recruitment of protein phosphatase 1 (PP1), which dephosphorylates the transporter thereby blocking the inhibition by the WNK/SPAK pathway (Yang et al., 2011; Lee et al., 2012). The autoinhibition by AID is stabilized by recruitment of SPAK by the WNK kinases (Hong et al., 2013). A staurosporine-sensitive kinase appears to be involved in the PIP_2_ mediated activation of NBCe1-B and -C (Thornell et al., 2012). The mechanism by which PIP_2_ activates NBCe1-A remains to be determined (Wu et al., 2009).

Intracellular Mg^2+^ inhibits bovine NBCe1-B currents heterologously expressed in HEK 293 cells, and the inhibition was decreased by truncation of NBCe1-B specific N-terminal residues (Yamaguchi and Ishikawa, 2008). Expression of IRBIT lowered the sensitivity of NBCe1-B currents to Mg^2+^ inhibition (Yamaguchi and Ishikawa, 2012) via an unknown mechanism. Mg^2+^ also inhibits the NBCe1-A variant that has been hypothesized to involve a Mg^2+^-dependent phosphatase (5'-lipid phosphatase) dephosphorylation of PIP_2_to PIP (Wu et al., 2009). Whether this effect is mediated by the NBCe1-A cytoplasmic N-terminus or C-terminal tail is unknown. The inhibition of NBCe1 variants by cytosolic Mg^2+^ has been postulated to provide a mechanism for reducing cellular dysfunction in ischemia (Wu et al., 2009).

Distal to the IRBIT interaction site in NBCe1-B, the Hsp70-like stress 70 protein chaperone STCH interacts with the residues amino acids 96–440 in the amino terminus and significantly increases the plasma membrane expression of the transporter (Bae et al., 2013). Whether the plasma membrane expression of NBCe1-A, -C, -D, and -E also increases after STCH binding (since this region is common to all known variants) has not been studied. Although the biologic role of this interaction has not been determined, it has been proposed that during acidemic conditions, enhanced NBCe1 transport would improve the efficiency of intracellular pH recovery (Bae et al., 2013).

## The transmembrane region (structure and role in ion transport)

NBCe1-A like all SLC4 proteins has a large transmembrane region that mediates ion transport. The current topology model of NBCe1-A indicates that it contains 14 transmembrane segments (TMs) with various lengths, which may resemble certain topologic characteristics with the vGLUT and LeuT prokaryotic Na^+^-coupled transporters (Yamashita et al., 2005; Watanabe et al., 2010; Zhu et al., 2010a,b). Topological analysis shows that NBCe1-A has a large glycosylated extracellular loop 3 (EL3) and a smaller loop 4 (EL4), and the extracellular surface is compactly folded. Various pRTA missense mutations reside in the transmembrane region thereby altering its structure-functional properties. The role of each TM in the structure-functional properties of NBCe1-A and the perturbations induced by specific pRTA mutations will now summarized in detail.

## TM1—pRTA S427L mutation; topological folding

Evidence for the importance of TM1 is derived from structure-function studies of the wild-type transporter and from experiments that have addressed the abnormalities caused by the TM1 S427L pRTA mutation (Dinour et al., 2004; Li et al., 2005; Zhu et al., 2009, 2010b, 2013a). NBCe1-A-TM1 has unique properties that have been detailed in recent structural studies (Zhu et al., 2009, 2010b, 2013a). Specifically, TM1 is longer than a standard TM and contains 31 amino acids with an N-terminal cytosolic portion that has a helical conformation connecting the plasma membrane portion and the remaining cytoplasmic portion. In addition, TM1 contains several key residues including Asp^416^, Gln^424^, Tyr^433^, and Asn^439^ whose substitution with cysteine causes intracellular retention likely due to protein misfolding. Ala^428^, Ala^435^, and Thr^442^ are functionally important residues that line the ion permeation pathway. Thr^442^ also forms part of an extracellular gate involved in ion entry to the permeation pathway.

The importance of TM1 is further highlighted by the S427L pRTA mutation which decreases transporter function by 90% coupled with inability to reverse the direction of transport at very negative membrane potentials (Dinour et al., 2004). Recent studies have shown that Ser^427^ resides in a space-confined region and that the hydrophobicity of the serine side chain plays an important role in helix packing (Zhu et al., 2013a). Accordingly, the S427L mutation alters the conformation of TM1 by abolishing the potential ionic interactions between helices resulting in collapsed or an altered configuration of NBCe1-A ion permeation pathway. When expressed in MDCK cells, the S427L mutant is preferentially mistargeted to the apical membrane providing an additional mechanism for inducing pRTA (Li et al., 2005).

## TM3—pRTA T485S and G486R mutations; NBCe1 electrogenicity

NBCe1-A-TM3 contains two adjacent mutations, T485S and R486R that cause pRTA (Horita et al., 2005; Suzuki et al., 2008, 2010; Zhu et al., 2010b, 2013b). The T485S mutation is unusual in that serine and threonine are similar structurally and chemically, and their substitution would not be expected to alter the function of the transporter. A recent study addressing this question has shown that Thr^485^ is located in an aqueous confined region accessible to NEM labeling that undergoes substrate-driven intra- and extracellular facing conformational changes (Zhu et al., 2013b). These results suggested that Thr^485^ resides in an NBCe1-A ion interaction site. This interpretation was further supported by the finding that the adjacent G486R mutation perturbs the function of the transporter and causes pRTA by altering the orientation of Thr^485^ (Zhu et al., 2013b).

**Figure 3:**
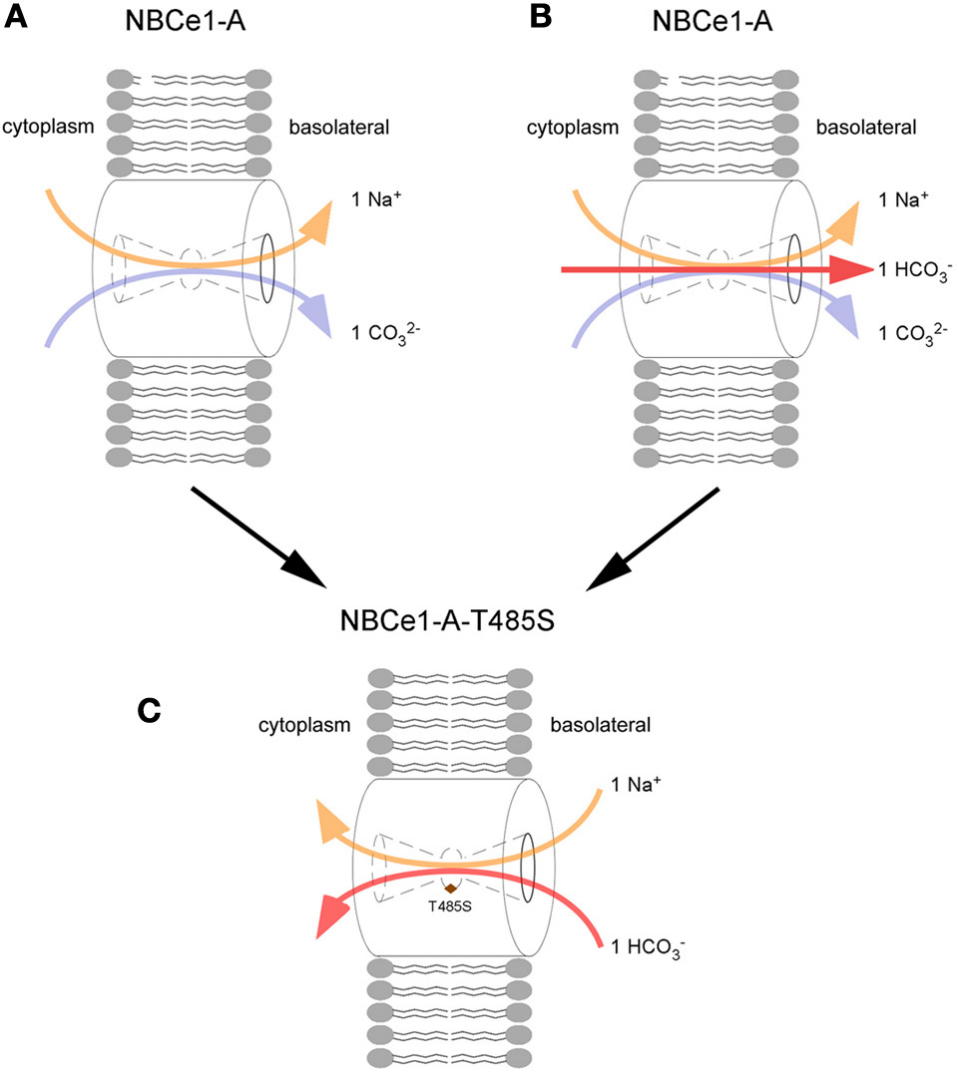
**Loss of electrogenicity caused by the T485S pRTA mutation**. The charge transport stoichiometry of NBCe1-A in the human proximal tubule is unknown and 2 potential models are depicted: In the 1:2 charge transport stoichiometry mode **(A)**, wt-NBCe1-A is modeled to lose its electrogenecity because the T485S mutant **(C)** preferentially transports Na^+^-HCO^−^_3_ rather Na^+^-CO^2-^_3_. i.e., the anion interaction site favors HCO^−^_3_ rather than CO^2−^_3_. **(B)** wt-NBCe1-A transporting 1 Na^+^ + 1 HCO^−^_3_ + 1 CO^2−^_3_ (1:3 charge transport stoichiometry), following the loss of CO^2−^_3_ interaction, the T485S mutant is converted into an electroneutral Na^+^-HCO^−^_3_ transporter **(C)**.

The effect of the T485S mutation on transporter function is complex. Base transport is decreased by ~50% (Horita et al., 2005; Suzuki et al., 2008, 2010; Zhu et al., 2010b, 2013b) and in addition, the mutant transporter is electroneutral because of a change in its charge transport stoichiometry (positive charge to negative charge transport ratio) (Zhu et al., 2013b). The mechanism of the change in charge transport stoichiometry has recently been elucidated. Experiments using NO^−^_3_ as a surrogate for CO^2−^_3_ have shown that human wt-NBCe1-A functioning with a charge transport stoichiometry of 1:2 in HEK 293 cells mediates electrogenic Na^+^-CO^2−^_3_ cotransport (Zhu et al., 2013b). Furthermore, the electroneutral T485S mutant fails to transport Na^+^-NO^−^_3_. To account for these finding two pRTA proximal tubule transport models have been proposed (Figure 3). In model 1, assuming wt-NBCe1-A normally has a 1:2 charge transport stoichiometry *in vivo*, the T485S mutant loses its electrogenecity because of the preferential transport of Na^+^-HCO^−^_3_ rather Na^+^-CO^2−^_3_. In model 2, assuming a wt-NBCe1-A charge transport stoichiometry of 1:3 *in vivo* (1 Na^+^ + 1 HCO^−^_3_ + 1 CO^2−^_3_ cotransport), the T485S mutant becomes electroneutral because of loss of CO^2−^_3_ interaction resulting in Na^+^-HCO^−^_3_ cotransport. Independent of the initial stoichoimetry, the T485S mutant differs from other pRTA mutations in that transepithelial bicarbonate transport would be predicted to be impaired by a fundamentally different mechanism: the basolateral cellular influx of Na^+^ and HCO^−^_3_ down their respective concentration gradients.

## TM4—pRTA R510H, W516X, and L522P mutations; ER retention and protein folding

The R510H and L522P pRTA mutations result in ER retention due to protein misfolding (Igarashi et al., 1999; Horita et al., 2005; Li et al., 2005; Demirci et al., 2006; Suzuki et al., 2008, 2010; Zhu et al., 2010b; Yamazaki et al., 2013). On this basis and because TM4 carries potential signal anchor and stop transfer sequences, it has been suggested that TM4 functions as a scaffolding helix that is essential for normal protein folding. Although not well studied, the finding that the histidine substitution at Arg^510^ leads to protein misfolding suggests the magnitude of the positive charge and/or the size of the side-chain of Arg^510^ is required for ionic interaction between TM4 and neighboring TMs. The L522P mutation has been more extensively studied where it has been shown that L522C and L522I are not ER retained and do not alter NBCe1-A plasma membrane processing (Zhu et al., 2010b; Yamazaki et al., 2013). These findings suggest that the proline likely as a result of increased flexibility causes helix disruption, significant protein misfolding, and subsequent intracellular retention. Finally W516X mutation located in TM4 results in a truncated and likely misfolded protein (Lo et al., 2011).

## TM5—pharmacologic inhibition; role of Asp^555^ in substrate selectivity

The stilbene inhibitor 4,4′-diisothiocyanatostilbene-2,2′-disulfonate (DIDS) binds to NBCe1-A from both the extracellular and intracellular surfaces. Extracellular DIDS binds to a KKMIK motif in TM5 resulting in inhibition of transporter function through a mechanism that is currently unknown (Lu and Boron, 2007). At more positive membrane voltages the apparent affinity of the interaction between DIDS and transporter is increased that has been attributed to membrane voltage dependent conformational changes (Yamaguchi and Ishikawa, 2005; Lu and Boron, 2007). The intracellular binding site(s) is currently undetermined (Heyer et al., 1999). Moreover, site(es) of the interaction of other inhibitors including tenidap (Ducoudret et al., 2001), benzamil (Ducoudret et al., 2001), and S0859 (Ch'en et al., 2008) and whether they interact with residues in TM5 remains to be addressed. In separate studies, the TM5- Asp^555^ residue that is near the proposed DIDS binding site has been identified as being functionally important and involved in anion selectivity (Yang et al., 2009). Substitution of Asp^555^ with glutamate induces outward rectifying Cl^−^, NO^−^_3_, and SCN^−^ currents.

## EL3 and EL4—disulfide formation; carbonic anhydrase binding; NBCe1 electrogenicity

EL3 (56 amino acids), the largest loop in NBCe1 is glycosylated and contains 4 cysteine residues (Zhu et al., 2012). Structural studies have shown that in each monomer, the 4 cysteines are intramolecular disufided forming a highly ordered topologically domain (Zhu et al., 2012). Based on homology with Cys-loop ligand-gated ion channel superfamily, this region in EL3 may bind a ligand that regulates the function of the transporter. The next extracellular loop, EL4, binds plasma membrane CAIV (Alvarez et al., 2003) and CAIX (Orlowski et al., 2012). EL4 which contains a large number of proline residues is also involved in the electrogenic properties of the transporter (Chen et al., 2011). It is possible that the flexible EL4 loop (formed between TMs7-8) interacts with residues embedded in the lipid bilayer that alter ion interaction with NBCe1-A. Residues in TM8 which potentially form part of the ion permeation pathway (see below) may interact with EL4 to modulate the electrogenic properties of NBCe1.

## TM8—Leu^750^; ion permeation pathway

Based on the finding that residues in TM8 were reported to form part of the ion permeation pathway in AE1 (Tang et al., 1999), McAlear et al. proposed that NBCe1-A-TM8 might have similar properties (McAlear and Bevensee, 2006). Using a cysteine scanning mutagenesis approach, residues in TM8 were identified to play a role in ion permeation with Leu^750^ being particularly involved. In further support of residues in TM8 being involved in ion permeation, pCMBS accessibility was decreased in the presence of substrate ions and the stilbene inhibitor 4.4-dinitro-stilbene-2,2'-disulfonate (DNDS).

## TM10—pRTA A799V mutation; hypokalemia and muscle weakness

Although more typically seen in patients with Fanconi's syndrome (Soriano, 2002), patients with isolated pRTA can be hypokalemic as a result of initial enhanced collecting duct HCO^−^_3_ delivery (prior to achieving a steady state), an elevation of serum aldosterone due to volume depletion, and enhanced collecting duct HCO^−^_3_ delivery during bicarbonate therapy. In addition to the known renal and extrarenal phenotype of patients with NBCe1 mutations, Deda et al reported a patient with extra-renal K^+^ loss (diarrhea, and vomiting) that resulting in severe acute hypokalemia (Deda et al., 2001). The patient was subsequently shown to have a new A799V pRTA mutation that significantly decreased the function of mutant NBCe1-A (Horita et al., 2005; Suzuki et al., 2010; Zhu et al., 2010b). Further studies on the NBCe1-A799V demonstrated that the mutant transporter had an associated HCO^−^_3_-independent cation leak conductance (Parker et al., 2012). It was hypothesized that since there is evidence that NBCe1 is expressed in skeletal muscles (sarcollema and possibly t-tubules), during severe hypokalemia, the HCO^−^_3_-independent cation leak conductance would result in exacerbated muscle weakness when compared to patients with other NBCe1 pRTA mutations.

## TM11-14—pRTA R881C mutation; ER retention and protein folding

The TM12 R881C pRTA mutation (Horita et al., 2005; Toye et al., 2006; Suzuki et al., 2010; Zhu et al., 2010a,b) induces ER retention when expressed in mammalian cells likely due to protein misfolding, suggesting that TM12 is involved in helix packing (Zhu et al., 2010a). In *Xenopus* oocytes where plasma membrane expression is decreased but present (Horita et al., 2005; Toye et al., 2006), when corrected for the plasma membrane expression level, the transporter appears to function normally (Toye et al., 2006).

Recent evidence suggests that the entire C-terminal transmembrane region from TM10-14 (Ala^800^ and Lys^967^), plays an important role helix packing and protein folding (Zhu et al., 2010a). Specifically, 18 residues clustered on the surface of each TM form intramolecular hydrogen bonds contributing to helix packing thereby stabilizing the structure of NBCe1. The loops connecting TMs11-14 are tightly folded rather than being aqueous exposed. At the beginning of TM11, 5 residues likely function as a topogenic signal guiding TM11 into the lipid bilayer. TM11 and 12 are abruptly bent into the lipid bilayer at Met^858^ which is bracketed by Pro857 and Pro858. Cryptic intracellular loop 6 (IL6) that connects TM12 and 13 is also tightly folded and appears to interact with the cytoplasmic region. TM12-Lys^924^ appears to act as a counter-ion countributing to helix packing. The final extracellular 7 (EL7; Thr^926^-Ala^929^) is only slightly exposed to the aqueous media, suggesting that it is folded in the transmembrane region.

It has also been hypothesized based on domain swapping experiments that TM12 and TM6 form a “functional unit” required for plasma membrane processing (Chen et al., 2012). Specifically, in an NBCe1-A chimera formed by replacing TM6 and what was referred to as “TM12” (according to the most recent topologic model of NBCe1 containing residues from TM12, intracellular loop 6 (IL6) and TM13) with corresponding regions from electroneutral NBCn1, membrane processing was significantly impaired. This interpretation of the data is likely premature given that mixed chimera proteins can be improperly folded resulting in ER retention (Fujinaga et al., 2003). In addition, specific residues in TM12 have been shown to play a role in helix packing (Zhu et al., 2010a).

## The C-terminal cytoplasmic tail—basolateral targeting sequence; pRTA 65 bp-del mutation; carbonic anhydrase binding

The intracellular lipid/aqueous interface of TM14 is marked at Asp^960^ and Pro^963^ likely induces a kink exposing the C-terminal cytoplasmic tail to the aqueous cytoplasm (Zhu et al., 2010a). In the NBCe1-A dimer, there is a close association between the C-terminal cytoplasmic tail of each monomer raising the question as to whether this grouping of two stretches of strongly charged amino acids plays an important functional regulatory role (Zhu et al., 2010c). Two pieces of evidence indicate that the C-terminal tail plays a role in membrane processing and targeting. First, in the pRTA 65 bp-del frame shift mutation which truncates the C-terminal tail, the mutant transporter is retained intracellularly in the ER in mammalian cells (though not in *Xenopus* oocytes) (Suzuki et al., 2010). Secondly, when expressed in MDCK cells, a C-terminal tail ^1010^QQPFLS^1015^ motif functions as a basolateral targeting signal (Li et al., 2004).

The C-terminal tail may also have a functional role. Using isothermal titration calorimetry CAII was shown to bind to the cytosolic C-terminus of NBCe1 at a D^986^NDD^989^ motif (Gross et al., 2002). Based on studies of CAII interaction with AE1, it was proposed that NBCe1 and CAII form a transport metabolon wherein NBCe1 transfers ions intra-molecularly to CAII. Subsequent studies provided additional support for a functional interaction between NBCe1 and CAII (Pushkin et al., 2004; Becker and Deitmer, 2007). In addition, other CA isoforms including plasma membrane CAIV and CAIX were also shown to bind to NBCe1-EL4 (Alvarez et al., 2003; Orlowski et al., 2012). However, not all groups were able to document a functional interaction with CAII (Lu et al., 2006; Piermarini et al., 2007; Yamada et al., 2011). In comparing the conflicting data between these studies, there is a need to consider the various techniques/preparations used including differences in aqueous binding vs. solid phase interaction, sensitivity, specificity, signal/noise, and artifacts introduced by the various assays employed. In support of a lack of clinically important interaction between CAII and NBCe1 is the finding that some patients with loss of function CAII mutations, and mice with targeted disruption of CAII do not have as severe defect in proximal tubule bicarbonate absorption as predicted (Sly et al., 1985; Lewis et al., 1988). In addition, the systemic acidemia due to loss of CAII is not as severe as the metabolic acidosis in patients with NBCe1 mutations and mice with loss of NBCe1 function. Whether compensatory mechanisms account for these findings and the potential utility of performing conditional targeted disruption of CAII in mice remains to be determined. A renal phenotype has also not been reported in patients with CAIV mutations with retinitis pigmentosa (RP17) (Rebello et al., 2004). The important question of whether carbonic anhydrase can bind and modulate the function of NBCe1 has recently been re-examined by Schueler who showed that CAI, CAII, and CAIII stimulate NBCe1-A transport in *Xenopus* oocytes (Schueler et al., 2011). The increase in NBCe1-A transport was attributed to CA enzymatic activity rather than an intramolecular proton shuttle reaction.

## Additional potential genetic causes of isolated pRTA

In 1977, a family from Costa Rica was reported of whom 9 members had isolated pRTA and short stature with dominant inheritance (Table 2; Luis et al., 1977). A follow-up study on 2 of the family members documented decreased bone density (Lemann et al., 2000). A second family with autosomal dominant isolated pRTA and short stature has been subsequently reported (Katzir et al., 2008). The authors ruled out mutations in the coding regions and splice sites of 9 proteins involved in proximal tubule H^+^/base transport including CAII, CAIV, CAXIV, NBCe1, NHE3, NHE8, NHERF1, and NHERF2, and PAT1(CFEX). Importantly, intron sequence and promoter abnormalities in the genes coding for these proteins were not completely excluded. Determining the molecular basis for the cause of pRTA in these patients will potentially uncover new transport and/or regulatory mechanisms involved in mammalian proximal tubule bicarbonate absorption.

## Additional thermodynamic considerations and charge transport stoichiometry

Thermodynamics dictates that when the electrochemical driving force (μ) across NBCe1-A in the basolateral membrane of the proximal tubule has a positive value, the transporter will mediate Na^+^-coupled base efflux in the direction of cytoplasm to peritubular blood (Kurtz et al., 2004; Zhu et al., 2013b). The charge transport stoichiometry of NBCe1 is an important determinant of the value of the electrochemical driving force. Although the charge transport stoichiometry of NBCe1-A in the human proximal tubule has not been measured, it has been implicitly assumed to be 1:3, largely based on the belief that a 1:2 charge transport stoichiometry would not result in a positive value for μ. The literature on the charge transport stoichiometry of NBCe1-A is somewhat confusing given that species-specific, preparation specific, cell-specific, and perhaps technique-specific factors may play a role. In the *in vivo* rat proximal tubule Yoshitomi et al obtained a value for the charge transport stoichiometry of 1:3 (Yoshitomi et al., 1985). In the isolated perfused rabbit proximal tubule, the charge transport stoichiometry appears to vary with the properties of the solutions used: i.e., 1:2 in Ringers solution (Seki et al., 1993) and 1:2.7 (Müller-Berger et al., 1997) in tubules bathed in norepinephrine and DMEM. In *Necturus* proximal tubules *in vivo*, the charge transport stoichiometry was found to decrease from 1:3 to 1:2 during respiratory acidosis (Planelles et al., 1993). In heterologously expressed NBCe1-A, Ca^2+^ (Müller-Berger et al., 2001), phosphorylation state, (Gross et al., 2001b) and cell-type (Gross et al., 2001a) appear to modulate the charge transport stoichiometry. Specifically, PKA dependent phosphorylation of NBCe1-A-Ser^982^ was reported to shift the charge transport stoichiometry from 1:3 to 1:2 (Gross et al., 2001b). It has been postulated that Ca^2+^ also induces a shift in charge transport stoichiometry from 1:2 to 1:3 via a change in the phosphorylation state the transporter (Müller-Berger et al., 2001). Whether Ca^2+^ modulation of the transporter stoichiometry can be detected in mammalian expression systems is unknown. Finally, whether NBCe1-A can change the stoichiometry in the *in vivo* human proximal tubule remains an unresolved question.

## Is NBCe1-A a Na^+^-Co^2−^_3_ cotransporter *in vivo?*

It is generally accepted that NBCe1-A functions as a Na^+^-CO^2−^_3_-HCO^−^_3_ cotransporter (charge transport stoichiometry of 1:3) thereby mediating Na^+^-coupled base efflux, despite the uncertainty as to the exact electrochemical driving force across the basolateral membrane in the human proximal tubule. An important unanswered question is whether NBCe1-A in the human proximal tubule has a charge transport stoichiometry of 1:2, as has been clearly demonstrated when the human transporter is heterologously expressed in mammalian HEK 293 cells (Zhu et al., 2013b). Although a charge transport stoichiomtery of 1:2 is compatible with a 1 Na^+^: 2 HCO^−^_3_ transport mode, recent data using NO^−^_3_ as a surrogate for CO^−^_3_ transport is more compatible with a Na^+^-CO^2−^_3_ cotransport mode (Zhu et al., 2013b). In addition, preliminary data using surface pH electrodes in the *Xenopus* oocyte expression system also suggests that rat NBCe1-A mediates Na^+^-CO^2−^_3_ cotransport (Lee et al., 2011). Unfortunately, given that the basolateral membrane potential and the *in vivo* gradients of the transported ions in the human proximal tubule are unknown, whether the electrochemical driving force is sufficient to drive NBCe1-A mediated basolateral Na^+^-coupled CO^2−^_3_ efflux is uncertain. Using data from the rat proximal tubule, a species where the necessary data is available, it has recently been calculated that NBCe1-A would indeed be capable of mediating basolateral Na^+^-CO^2−^_3_ efflux while at the same time being sensitive to small changes in the electrochemical chemical potential of the transported ions (Zhu et al., 2013b). If indeed future studies confirm that NBCe1-A normally functions in the proximal as a Na^+^-CO^2−^_3_ cotransporter, one could argue that its name should be changed for example to NCCe1-A (sodium carbonate cotransporter electrogenic 1-A).

In this regard, it is instructive to again consider how the loss of electrogenicity in the context of the T485S mutation causes pRTA. Unlike all other known pRTA mutations, NBCe1-A-T485S would be insensitive to the basolateral membrane potential. As shown in Figure 3, given the inwardly directed initial chemical gradients (peritubular to cell), the mutant transporter would be predicted to transport Na^+^-HCO^−^_3_ intracellularly across the basolateral membrane impairing proximal tubule HCO^−^_3_ absorption. If this is in fact is what is occurring *in vivo*, it would represent an entirely novel mechanism for generating pRTA. In this regard, transgenic mice expressing NBCe1-A-T485S would be an important tool for studying various aspects of this sequence of events in greater detail.

### Conflict of interest statement

The authors declare that the research was conducted in the absence of any commercial or financial relationships that could be construed as a potential conflict of interest.
